# Switching the NIR upconversion of nanoparticles for the orthogonal activation of photoacoustic imaging and phototherapy

**DOI:** 10.1038/s41467-022-30713-w

**Published:** 2022-06-07

**Authors:** Yang Yang, Jinshu Huang, Wei Wei, Qin Zeng, Xipeng Li, Da Xing, Bo Zhou, Tao Zhang

**Affiliations:** 1 MOE Key Laboratory of Laser Life Science & Institute of Laser Life Science, Guangdong Provincial Key Laboratory of Laser Life Science, College of Biophotonics, South China Normal University, Guangzhou, 510631 China; 2 State Key Laboratory of Luminescent Materials and Devices, South China University of Technology, Guangzhou, 510641 China; 3 Guangzhou Key Laboratory of Spectral Analysis & Functional Probes, College of Biophotonics, South China Normal University, Guangzhou, 510631 China

**Keywords:** Cancer, Optics and photonics, Nanoscience and technology, Materials science

## Abstract

Phototheranostics based on upconversion nanoparticles (UCNPs) offer the integration of imaging diagnostics and phototherapeutics. However, the programmable control of the photoactivation of imaging and therapy with minimum side effects is challenging due to the lack of ideal switchable UCNPs agents. Here we demonstrate a facile strategy to switch the near infrared emission at 800 nm from rationally designed UCNPs by modulating the irradiation laser into pulse output. We further synthesize a theranostic nanoagent by combining with a photosensitizer and a photoabsorbing agent assembled on the UCNPs. The orthogonal activation of in vivo photoacoustic imaging and photodynamic therapy can be achieved by altering the excitation modes from pulse to continuous-wave output upon a single 980 nm laser. No obvious harmful effects during photoexcitation was identified, suggesting their use for long-term imaging-guidance and phototherapy. This work provides an approach to the orthogonal activation of imaging diagnostics and photodynamic therapeutics.

## Abstract (web summary)

Upconversion nanoparticles can be used to both image and treat tumors. Here, the authors describe an upconversion nanoparticle that can switch from imaging to phototherapy by altering the NIR emission.

## Introduction

Cancer phototheranostics, an emerging combinatorial modality of light-diagnostics and -therapeutics, has been developed as personalized precision medicine to enhance the treatment accuracy and efficiency of conventional phototherapies particularly the representative photodynamic therapy (PDT)^1–7^. The clinical-approved PDT methodology refers to the photoactivation of a photosensitizer in the presence of molecular oxygen to generate reactive oxygen species (ROS) for destruction of pathological tissues^8–10^. To date, many efforts have been devoted to the photodynamic theranostics by developing functional photosensitizers to serve as both an imaging agent and a therapeutic agent^11–13^. However, in light of the limitations of currently available photosensitizers with synchronous activation of both diagnostic and therapeutic signals, the long-term and real-time diagnostic imaging may also induce unnecessary phototoxicity (including photooxidation by ROS and the photo-induced hyperthermia effect) in the normal tissues^14–16^. To meet the demand for precision medicine against cancers, programmable photoactivation of the photosensitizers for the safe imaging and efficient therapy in an orthogonal manner is highly desirable. However, it is still challenging due to the lack of ideal photoswitchable agents.

Among the reported orthogonal theranostic agents, the lanthanide-doped upconversion nanoparticles (UCNPs) with multiple wavelength-tunable emissions in visible or near-infrared (NIR) region have been demonstrated to be a remarkable candidate for the development of photoswitchable agents^17–22^. Some excitation-emission orthogonal UCNPs-based platforms were constructed for the programmable activation of cancer diagnosis and therapy^22–28^. However, in previous studies, two (or more) excitation laser wavelengths (e.g., 980 and 808 nm) and exquisite agents with core-multi-shell structure designs or different components are indispensable to achieve the switchable phototheranostics^29–31^. This makes the manufacturing process quite complicated and time-consuming, and the instruments are also complex. Moreover, the tunable upconverting emissions mainly locate in the UV-visible region, such as UV/blue^32^, green/red (or blue)^33,34^ and blue/green/red light^17,27,35,36^. The switchable NIR emission is still not achievable, which is believed to be more significant in the phototheranostics.

Interestingly, photon upconversion is a multi-step anti-Stokes process and each step may have a temporal characteristic. This means that some upconversion processes would be sensitive to the excitation duration, consequently generating tunable emission profiles as evident in the full-color emissive nanoparticles upon a combination of pulse laser excitation^17^. However, it should be noted that such orthogonal upconversion needs complex core-multi-shell nanoparticles which produce great difficulties in the experimental synthesis^22^. By contrast, we recently constructed a simple energy-migratory core-shell nanostructure toward red/green color switchable output by temporal manipulation of the Er^3+^–Yb^3+^ interactions under 980 nm single-wavelength excitation^37^. Moreover, the tunable NIR upconversion luminescence would be also available by employing suitable transitions in specific lanthanide ions. This result provides new chances for the switchable NIR upconversion in a much simpler nanostructure through tuning the pulse widths of a single-wavelength excitation, showing a remarkable advantage for bioimaging and phototherapy.

In this study, we report a facile strategy to programmably switch the 800 nm NIR emission in Yb/Tm/Er codoped UCNPs by precisely tuning the pulse width of 980 nm excitation laser to achieve the orthogonal real-time safe photoacoustic (PA) imaging and effective photodynamic therapy. We discover that the NIR emission of Tm^3+^ at 800 nm in such UCNPs is very sensitive to the excitation laser pulse width. This design markedly reduces the complex core-multi-shell nanostructure with multiple components as well as the synthetic procedure. We further construct an upconversion nanoagent (UCNPs-DI) by surface anchoring a diketopyrrolopyrrole polymer (DPP) dye with absorption covering the visible emission of UCNPs and the NIR-responsive photosensitizer of indocyanine green (ICG) dye. Under the excitation of short-pulse 980 nm laser, the ICG is not activated but the visible emissions of UCNPs can be captured by DPP to generate intense PA signals with no photodynamic and photothermal effect, which facilitates the long-term and real-time imaging of the in vivo tumors without significant phototoxicity observed. Under the continuous-wave (CW) 980 nm excitation, the switch-on NIR upconversion emission could efficiently activate the PDT effect to afford accurate and effective inhibition of the in vivo tumor growth. Therefore, such nanoagent can avoid the harmful effect during photoactivated imaging diagnosis and achieve efficient programmable and orthogonal phototherapy by regulating the laser pulse output upon the single 980 nm irradiation, further highlighting the bioapplications of non-steady-state mediated upconversion nanomaterials.

## Results

### Mechanistic design of UCNPs with switchable NIR emission

**Fig. 1:**
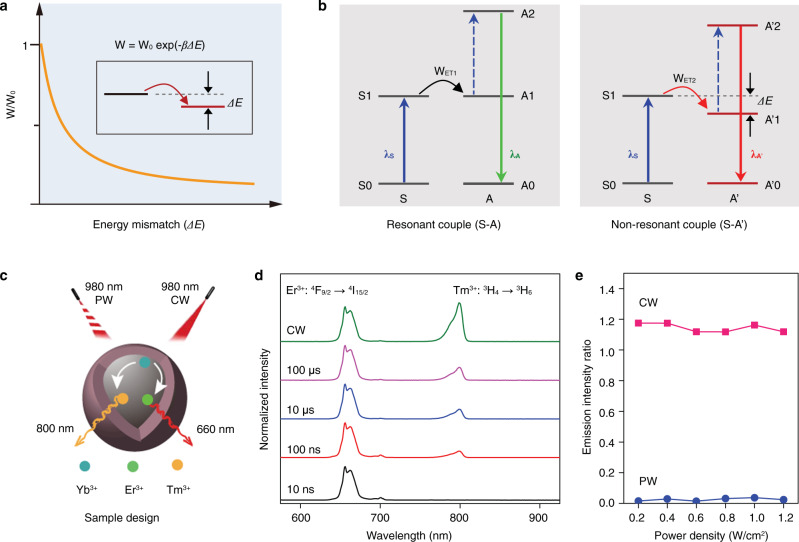
Mechanistic design towards NIR switchable upconversion. **a** Schematic of energy transfer rate (W) depending on the energy mismatch (*ΔE*) between the relevant energy levels of sensitizer (S) and activator (A). Inset shows the non-resonant energy transfer from S to A. Note that W_0_ is the rate when *ΔE* = 0, and *β* presents a positive parameter. **b** Comparison of the resonant (left) and non-resonant (right) energy transfer upconversion in sensitizer-activator coupled systems. **c** Schematic of the orthogonal emissive properties of the specially designed UCNPs, which show red/NIR emissions with 980 nm CW laser excitation and the NIR emission vanishes with short pulse 980 nm PW laser excitation. **d** Upconversion emission spectra of the UCNPs under CW 980 nm excitation and PW 980 nm excitation with a reduction of the pulse width. **e** Dependence of the NIR-to-red emission intensity ratio of the UCNPs on pump power density upon CW and PW (with width of 10 ns) 980 nm laser.

In general, the energy transfer rate in a given sensitizer-activator codoped system depends closely on the energy matching between the emission of sensitizer and the absorption of activator, which shows a decline following an exponential function of the energy gap (Fig. 1a). The upconversion system with an energy mismatch would result in a relatively slow upconversion process than that of the resonantly coupled system (Fig. 1b), allowing to filtrate such upconversion luminescence by reducing the pulse duration time of the excitation laser. Therefore, the dynamic control of switchable upconversion performance in much simpler structure nanoparticles becomes highly desirable.

As a proof-of-concept, we select the Yb^3+^–Tm^3+^ couple for the NIR upconversion and the Yb^3+^–Er^3+^ couple for the red upconversion. Note that there is an energy mismatch between Yb^3+^ (^2^F_5/2_) and Tm^3+^ (^3^H_5_), while it is resonant for the Yb^3+^ (^2^F_5/2_) and Er^3+^ (^4^I_11/2_). The NaYF_4_:20%Yb,1.5%Er,0.3%Tm nanoparticles were prepared for the switchable red/NIR luminescence under CW and pulsed-wave (PW) excitations (Fig. 1c). These UCNPs show uniform nanoparticles with an average diameter of ∼26 nm according to the transmission electron microscopy (TEM) images (Supplementary Fig. 1a). The d-spacing of ~0.323 nm calculated by fast Fourier transform (FFT) analysis corresponds to the (111) crystal plane of hexagonal NaYF_4_ (Supplementary Fig. 1b), which is in agreement with the selected-area electron diffraction pattern (Supplementary Fig. 1c) and X-ray diffraction (XRD) pattern (Supplementary Fig. 2). Figure 1d shows the upconversion emission spectra under 980 nm excitation. As expected, the red emission of Er^3+^ at 660 nm from its ^4^F_9/2_ → ^4^I_15/2_ transition and the NIR emission of Tm^3+^ at 800 nm from its ^3^H_4_ → ^3^H_6_ transition were clearly observed under the CW 980 nm excitation. Intriguingly, upon the PW 980 nm laser, the UCNPs show a gradual decrease in the NIR emission with reducing the pulse width, and no NIR emission can be observed when the pulse width reaches to 10 ns. Moreover, such NIR-switchable feature can preserve over a large range of pump power densities (Fig. 1e and Supplementary Fig. 3). These observations quantify the UCNPs an ideal NIR photoswitchable candidate to build the orthogonal theranostic agents.

**Fig. 2:**
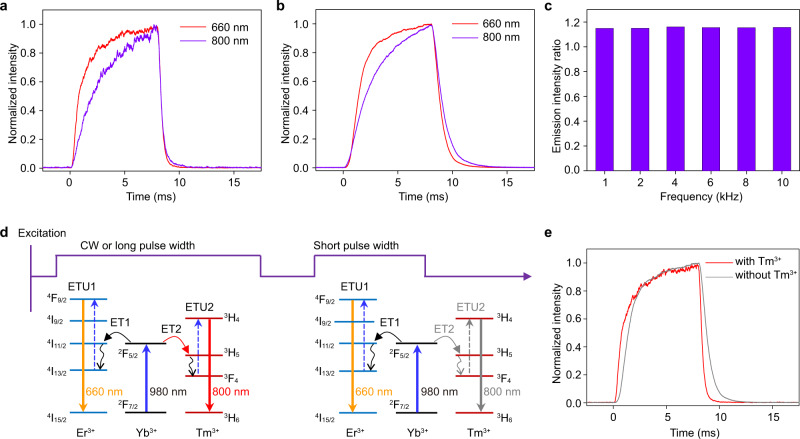
Switchable NIR upconversion mechanism through non-steady-state excitation. **a** Time-dependent upconversion emission profiles of Er^3+^ at 660 nm and Tm^3+^ at 800 nm for the UCNPs under PW 980 nm excitation with a duration time of 8 ms. **b** Time-dependent upconversion emission profiles of Er^3+^ at 660 nm and Tm^3+^ at 800 nm for the UCNPs doping with only Er^3+^ and Tm^3+^, respectively, under PW 980 nm excitation with a duration time of 8 ms. **c** NIR-to-red emission intensity ratios under different excitation frequencies for the UCNPs. **d** Schematic dynamic control of upconversion in Yb^3+^/Er^3+^/Tm^3+^ codoped system for 660 nm red and switchable 800 nm NIR emissions under 980 nm excitation with CW or PW laser. **e** Comparison of the time-dependent upconversion emission profiles of Er^3+^ at 660 nm for the UCNPs with and without codoping of Tm^3+^ under 980 nm excitation with a duration time of 8 ms.

To further examine the non-steady-state upconversion mechanism, we measured the time-dependent upconversion emission profiles at 660 and 800 nm, respectively. As displayed in Fig. 2a, the red upconversion emission exhibits a faster rise time than that of the NIR emission before reaching the steady-state. This observation suggests that the upconversion of the NIR emission of Tm^3+^ indeed needs more time by contrast to the red emission originating from the non-resonant energy transfer from Yb^3+^ (^2^F_5/2_) to Tm^3+^ (^3^H_5_). Consequently, the NIR to red emission intensity ratio shows a monotonous decline with the time during the excitation pulse. Hence, reducing the pulse width of the excitation laser becomes a facial but effective way to switch off the NIR emission. It should be noted that the co-doping of Er^3+^ and Tm^3+^ into NaYF_4_ lattice imposes no impact on their rise time feature (Fig. 2b), which confirms the dynamic manipulation of the NIR switchable output. The upconversion performance is independent on the excitation frequencies (Fig. 2c and Supplementary Fig. 4), which is useful for biological application. We also examined the influence of Tm^3+^ content on the energy transfer process by preparing the control UCNPs with Tm^3+^ from 0.3% to 1.5%. It can be found that increasing the concentration of Tm^3+^ in the UCNPs only results in a slight change in the rise time of NIR emission, which is always slower than that of the red upconversion emission (Supplementary Fig. 5). The total energy transfer processes during the dynamic control of the excitation are schematically illustrated in Fig. 2d. Another merit lies in that the presence of Tm^3+^ can promote the red upconversion of Er^3+^ slightly through the energy circling of Er^3+^ (^4^I_11/2_) → Tm^3+^ (^3^H_5_) → Er^3+^ (^4^I_13/2_) (Supplementary Fig. 6). This is consistent with the faster rise time of the red emission of the UCNPs after codoping of Tm^3+^ (Fig. 2e). Taken together, the results clearly confirm the validity of using the non-steady-state excitation towards the remarkable NIR-switchable effect of UCNPs, which has never previously been reported in the lanthanide-based nanoparticles. In the next, the pulse laser with width of 10 ns and frequency of 10 Hz was selected for the phototheranostics investigation.

### Construction and characterization of orthogonal phototheranostic nanoagent

**Fig. 3:**
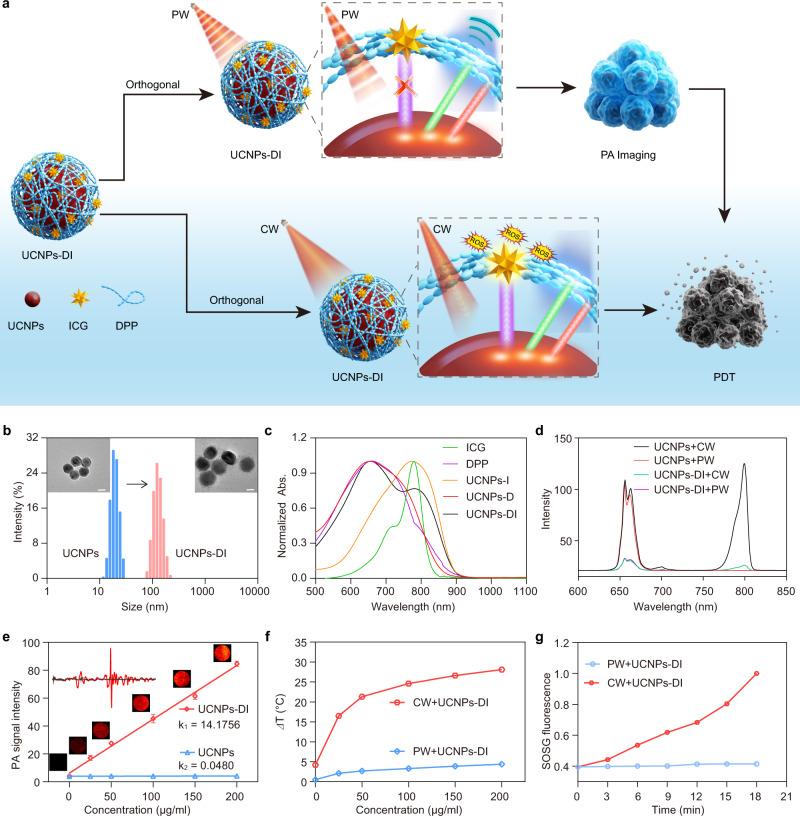
Design and characterization of the orthogonal UCNPs-DI nanoagent. **a** Schematic showing the orthogonal regulation of UCNPs-DI for photoacoustic imaging-guided on-demand treatment. **b** TEM images and DLS of UCNPs and UCNPs-DI, respectively. Scale bar, 20 nm. **c** Absorption spectra of ICG, DPP, UCNPs-I, UCNPs-D, and UCNPs-DI. **d** Upconversion emission spectra of UCNPs and UCNPs-DI under 980 nm PW or CW irradiation. **e** PA intensity of UCNPs-DI as a function of concentration (*R*^2^ = 0.97789), and the inset shows the point PA signal. Data are presented as mean ± SD (*n* = 3). **f** Temperature changes of UCNPs-DI under irradiation of 980 nm PW or CW laser at the same power density (0.5 W/cm^2^). **g** Normalized SOSG fluorescence intensity in the presence of UCNPs-DI under 980 nm PW or CW irradiation.

To demonstrate the dynamic control of the orthogonal photodiagnostics and phototherapeutics for the safe imaging-guided on-demand phototherapy, the nanoagent UCNPs-DI was synthesized via assembly with a visible-absorbing polymer dye DPP and the NIR-activatable photosensitizing dye ICG^38,39^ on the surface of UCNPs (Fig. 3a and Supplementary Fig. 7). The as-prepared DPP has broadband absorption (550–700 nm), high optical absorption extinction coefficient (46.8 L/(g cm)), and high photothermal conversion efficiency (Supplementary Fig. 8, 48.4%), which enable effective energy transfer from UCNPs to DPP to generate PA signal via pulsed photoirradiation of the nanoagent^40,41^. In view of the typical NIR absorption of ICG at around 800 nm and the NIR-switchable emission of UCNPs, the photodynamic effect is non-responsive to the short-pulse irradiation of UCNPs-DI, facilitating the safe long-term and real-time PA imaging. Upon 980 nm CW irradiation, the switching-on NIR emission could activate the ICG to efficiently produce the cytotoxic ROS for phototherapy. Therefore, the orthogonal control of the designed UCNPs-DI under programmable photoactivation guarantees the safety of imaging and the precision of therapy.

Figure 3b shows dynamic light scattering (DLS) results and TEM images of UCNPs and UCNPs-DI. On the basis of DLS results, all samples present a narrow size distribution with peaks centered at 26 and 106 nm for UCNPs and UCNPs-DI respectively, confirming a good stabilization of UCNPs and UCNPs-DI. Then, the UCNPs and UCNPs-DI nanoagent seen in TEM could accord with DLS results. Figure 3c presents the absorption spectra of UCNPs-DI and the other controls, including DPP, ICG, UCNPs-D (containing only the DPP dye on the UCNPs), and UCNPs-I (containing only the ICG dye on the UCNPs). According to the absorption peaks recorded at 660 and 800 nm and the Fourier-transform infrared (FTIR) spectroscopy (Supplementary Fig. 9) of UCNPs-DI, the DPP and ICG were loaded on the nanoagent successfully. The luminescence spectra of UCNPs-DI together with the starting UCNPs were then recorded under the excitation of 980 nm PW/CW laser. As illustrated in Fig. 3d and Supplementary Fig. 10, compared with the starting UCNPs, the visible and NIR emission of UCNPs-DI under CW irradiation was suppressed dramatically due to the energy transfer from UCNPs to DPP and ICG, respectively. This was also characterized by the lifetime decline of the upconversion emission at 800 nm (from 463 to 293 μs, Supplementary Fig. 11)^42^. These results confirmed the successful construction of the aimed phototheranostic nanoagent UCNPs-DI.

The stability of the nanoagent in aqueous physiological environments was then investigated by monitoring the absorption changes of UCNPs-DI in different content of blood serum and in serum with different standing times (Supplementary Fig. 12)^43^. The absorption of UCNPs-DI showed no obvious changes under these conditions. Adjusting the pH scope of the PBS buffer solution from 5.0 to 8.5 also did not affect its absorption and the upconversion emission under both 980 nm PW and CW excitations (Supplementary Figs. 13, 14). The results show that UCNPs-DI has an excellent consistency in the physiological environments.

To verify the feasibility of UCNPs-DI in orthogonal PA imaging and photodynamic therapy with programmable excitations of 980 nm PW/CW laser, we then evaluated its performance in the photoinduced generation of acoustic effect and singlet oxygen (^1^O_2_) in aqueous solutions. Compared with the starting UCNPs, as depicted in Fig. 3e, we observed an intense concentration-dependent PA signal for the UCNPs-DI under 980 nm PW excitation, which is comparable with that of ICG under 800 nm PW excitation (Supplementary Fig. 15). The result also revealed that the PA effect generated by UCNPs-DI via the upconverted optical excitation is intense enough for PA tomography^44,45^. The pulsed irradiation of UCNPs-DI had no obvious photothermal effect, while the temperature of UCNPs-DI solution increased dramatically after exposing to the CW irradiation (0.5 W/cm^2^), which suggests that the UCNPs-DI would not induce hyperthermia damage during the photoacoustic diagnosis (Fig. 3f and Supplementary Fig. 16)^46^. The high photostability of UCNPs-DI was confirmed by 10 min photoirradiation of the sample with 5 cycles (Supplementary Fig. 16b). The ^1^O_2_ photogeneration capability of UCNPs-DI was then evaluated using the singlet oxygen sensor green (SOSG) as an indicator^47^. Figure 3g and Supplementary Fig. 17a demonstrated that almost no change was observed for the fluorescence of SOSG at 526 nm in the UCNPs-DI solution with 980 nm PW irradiation (10 ns), revealing no obvious photodynamic toxicity during the photoacoustic diagnosis. On the contrary, 980 nm CW irradiation of UCNPs-DI can generate abundant ^1^O_2_, in which the fluorescence of SOSG increased rapidly within 18 min (Supplementary Fig. 17b).

In view of the NIR light-absorbing characteristic of ICG, we also examined the contribution of ICG in UCNPs-DI to the photogenerated PA signal and ^1^O_2_. The results revealed that the absorption of free ICG at 980 nm was negligible no matter in its monomer, H-aggregated or J-aggregated form (Supplementary Fig. 18). The absorption of UCNPs-DI was very stable under the mimic physiological environment while it was negligible at 980 nm (Supplementary Fig. 19). Under the excitation of 980 nm PW laser, intense and comparable PA signals were recorded for the UCNPs-DI and UCNPs-D, while they were very weak for UCNPs, DPP, free ICG, and UCNPs-I (Supplementary Fig. 20). As a control, no obvious ^1^O_2_ generation was observed by direct CW irritation of the free ICG at 980 nm (Supplementary Fig. 21). Therefore, we confirm that the PA signal and ^1^O_2_ in UCNPs-DI solution was generated through the upconversion process instead of a direct excitation of the ICG at 980 nm irradiation.

The above results clearly indicate that the UCNPs-DI produced intense PA signals with negligible photothermal and photodynamic effect upon 980 nm PW irradiation, providing robust ex-vitro evidence for the feasibility of UCNPs-DI as a safe PA imaging candidate for long-time and real-time diagnosis or monitoring the therapeutic treatments. Moreover, switching the 980 nm laser from PW into CW modulation could significantly activate its photodynamic effect for the subsequent therapy. Overall, the above results provide substantial support for us to achieve photoacoustic imaging and photodynamic therapy in an orthogonal manner through programmable excitation of UCNPs-DI by PW/CW 980 nm laser.

### Orthogonal activation of cellular photodynamic effect

**Fig. 4:**
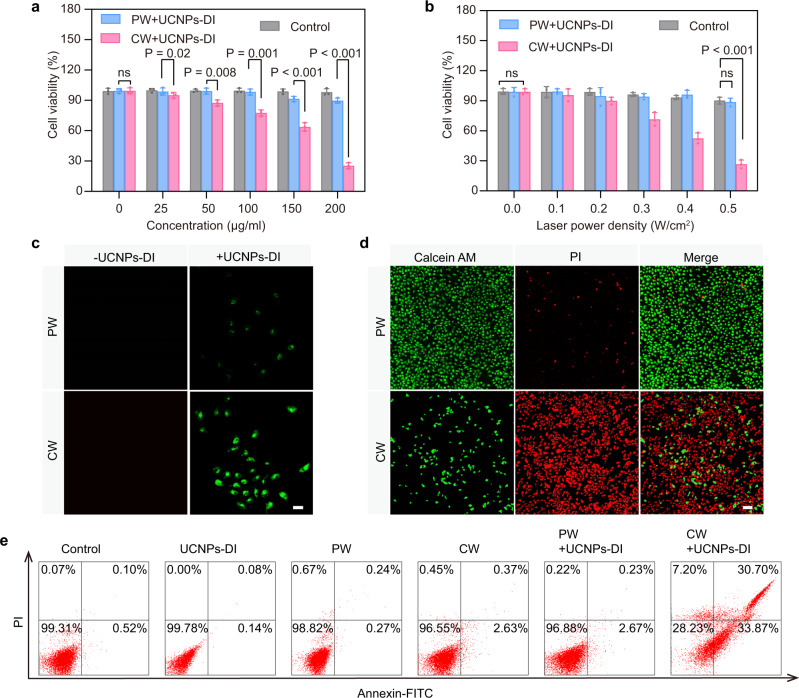
Cytotoxicity of UCNPs-DI in the orthogonal excitation. **a** MCF7 cells were incubated with different concentrations of UCNPs-DI for 4 h, then cells were irradiated with a 980 nm PW laser (10 ns, 0.5 W/cm^2^) or CW laser (0.5 W/cm^2^) for 3 min. The relative viabilities of MCF7 cells were determined by the standard MTT assay. **b** Relative viabilities of cells pretreated UCNPs-DI (200 μg/mL, 4 h) recorded under different laser power density. **c** Intracellular ^1^O_2_ generation detected in SOSG-stained MCF7 cells with different treatments. Scale bar, 50 μm. **d** Confocal fluorescence microscope images of calcein AM and PI co-stained MCF7 cells after various treatments indicated. Green and red colors represented live and dead cells, respectively. Scale bar, 100 μm. **e** Flow cytometric analysis of MCF7 cells death after different treatments. In **c**–**e**, the nanoagent was used to treat the cells for 4 h at the concentration of 200 μg/mL and the 980 nm PW (10 ns) or CW laser was employed to irradiate the cells for 3 min at the power density of 0.5 W/cm^2^. Mean values and error bars are defined as mean and SD, respectively. All data in **a** and **b** are presented as mean ± SD (*n*  =  3). Statistical differences *p* values were calculated by Student’s two-sided t-test (^ns^*P* > 0.05).

The cellular cytotoxicity of the UCNPs-DI with 980 nm PW and CW irradiation was firstly investigated in detail on human non-small cell human breast adenocarcinoma cancer cells (MCF7). The low dark cytotoxicity of the nanoagent was observed by co-incubation with MCF7 cells using the standard MTT assay (Supplementary Fig. 22). Figure 4a presents the viability of MCF7 cells treated with UCNPs-DI in different concentrations with irradiation of 980 nm PW (0.5 W/cm^2^) and CW (0.5 W/cm^2^) laser. Significantly, PW irradiation of the cells pretreated with UCNPs-DI did not induce obvious decrease in the cell viability. By contrast, it showed an evident drop in the cell viability under CW excitation and dropped to 28% when incubated with 200 μg/mL UCNPs-DI. At this concentration, as shown in Fig. 4b, light dose-dependent cell-killing effect of UCNPs-DI after incubated for 4 h was recorded when exposure to CW irradiation. The cell viability was inhibited dramatically to 27.2% with CW irradiation for 3 min.

The intracellular ROS non-generation in the PW + UCNPs-DI group and ROS generation in the CW + UCNPs-DI group were also confirmed using confocal laser scanning microscope (CLSM) as shown in Fig. 4c, which is consistent with the ROS detection results in solutions. In addition, the cell viability results were also verified by calcein AM (live cells staining; green) and propidium iodide (PI, dead cells staining; red) double-staining, respectively (Fig. 4d). The fluorescence imaging results showed that the UCNPs-DI has negligible cytotoxicity under PW irradiation but high photodynamic cytotoxicity under CW irradiation. We then further evaluated the cell damage mechanism of UCNPs-DI with PW/CW irradiation using flow cytometry with Annexin V-fluorescein isothiocyanate (V-FITC)/PI staining. The data revealed that in the cells treated with UCNPs-DI and PW irradiation, 2.67% of cells were stained with Annexin V-FITC and 0.23% of cells were stained with PI, suggesting that most cells were undamaged under this condition (Fig. 4e and Supplementary Fig. 26). The measurement taken at the group with UCNPs-DI after 980 nm CW irradiation showed that 33.87% of the cells were stained with FITC and 30.70% of the cells were stained with PI, suggesting that the UCNPs-DI with CW treatment caused obvious membrane damage and massive cell death. In addition, the UCNPs-DI was also found with efficient photo-inhibitory effect to other types of tumor cells (HepG2 and EMT6 cells) but with negligible toxicity to the normal LO2 cells (Supplementary Fig. 23). The above results confirmed the evidential potentials of UCNPs-DI as safe photoacoustic imaging candidate. Moreover, the results of UCNPs-DI under the 980 nm CW irradiation have validated the nanoagent as a photoswitchable agent for the effective phototherapy.

### In vivo photoacoustic imaging of tumor

**Fig. 5:**
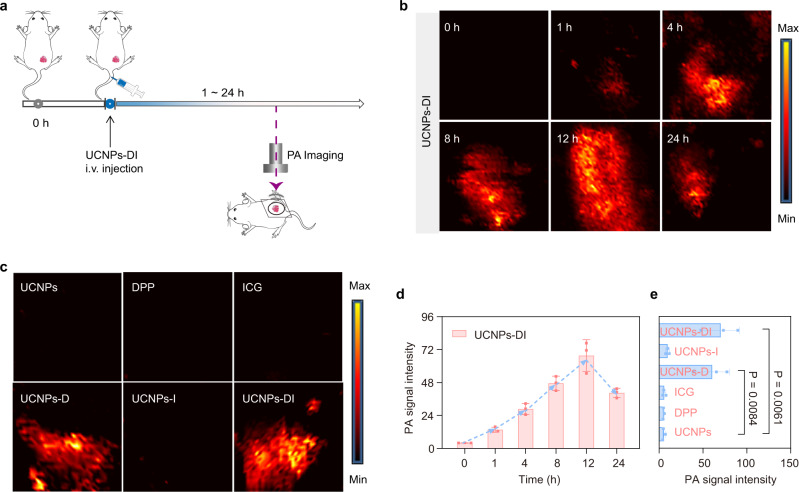
In vivo photoacoustic imaging of UCNPs-DI. **a** Schematic illustration of photoacoustic imaging of UCNPs-DI. **b** Real-time photoacoustic images of the tumors after systematic administration of UCNPs-DI in mice under irradiation of 980 nm PW laser. **c** Photoacoustic images of the tumors after systematic administration of UCNPs, DPP, ICG, UCNPs-D, UCNPs-I, and UCNPs-DI at 12 h intravenous post-injection under irradiation of 980 nm PW laser. **d** Quantitation of photoacoustic signals at the tumor sites of UCNPs-DI. **e** Quantitation of photoacoustic signals at the tumor sites of groups depictured in (**c**). Mean values and error bars are defined as mean and SD, respectively. All data in **d** and **e** are presented as mean ± SD (*n* = 3). Statistical differences *p* values were calculated by Student’s two-sided t-test.

With the intense PA effect and controllable-phototoxicity of UCNPs-DI validated in vitro and in living cells, we then investigated its performance in the real-time photoacoustic imaging of in vivo localized tumors (Fig. 5a). The PA imaging is a promising diagnostic tomography to monitor the molecular distribution and tumor size/morphology for therapeutical guidance due to its high resolution and noninvasive visualization of tissue structures^48^. After intravenous injection of UCNPs-DI or ICG into the EMT6 tumor-bearing mice, the PA images of tumor region were recorded at post-injection time of 1, 4, 8, 12, and 24 h, respectively. As depicted in Supplementary Fig. 24, no obvious enhanced PA signals over background were observed over time in the free ICG cohort under the pulsed 980 nm excitation. In significant contrast, the PA signal in the tumor region of the UCNPs-DI pretreated cohort started to be detectable at 4 h post-injection and gradually increased (Fig. 5b). The PA signal intensity reached the maximum at 12 h post-injection and maintained a relative high level after injection of 24 h (Fig. 5d). The PA signal in the UCNPs-DI cohort at 12 h post-injection was determined as 18.7 times greater than that in the UCNPs group (Fig. 5e). Moreover, intense and comparable PA signals were recorded in the UCNPs-DI and UCNPs-D cohort (Fig. 5c), which was consistent with the absorption and PA monitoring findings in vitro. Taken together, these results indicate that UCNPs-DI provide real-time PA imaging for definition of the tumor region and precise guidance for the subsequent phototherapy.

### Orthogonally regulated tumor imaging and phototherapy in vivo

**Fig. 6:**
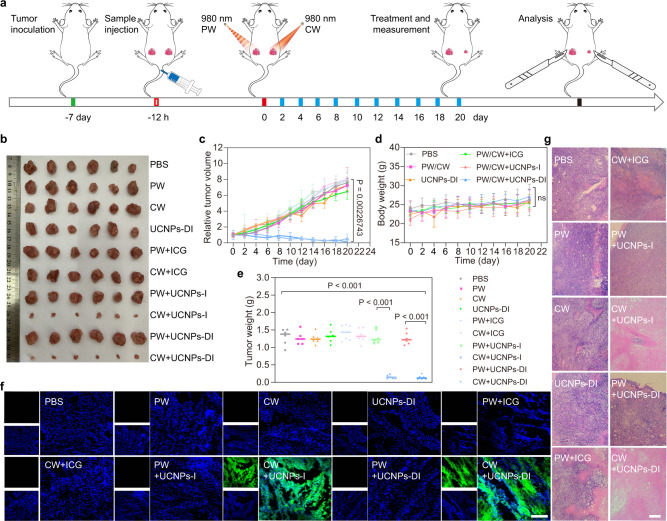
Orthogonally regulated tumor photo-treatment. **a** Schematic illustration of the PDT treatment regimen. **b** Photographs of tumor tissues extracted from the mice that received a set of treatments at 21 days including PBS, PW, CW, UCNPs-DI, PW + ICG, CW + ICG, PW + UCNPs-I, CW + UCNPs-I, PW + UCNPs-DI, and CW + UCNPs-DI. Relative tumor volume growth curve (**c**), mice body weight (**d**), and tumor weight (**e**) of the mice treated with different treatments. **f** SOSG immunofluorescence sections treated in series of groups. Scale bar, 100 μm. **g** H&E staining of tumor slices for treatments. Scale bar, 100 μm. All quantitative data in (**c**–**e**) are expressed as mean ± SD (*n* = 6, biologically independent mice). *P* values were calculated by Student’s two-sided t-test (^ns^*P* > 0.05).

We further evaluated the photoacoustic imaging-guided on-demand PDT efficacy in vivo by using the EMT6 tumor-bearing mice as the animal model (Fig. 6a). The mice were randomly divided into ten groups. The control group was injected with PBS; three groups received laser (PW/CW, 980 nm, 0.5 W/cm^2^ for 10 min) or UCNPs-DI intravenous injection alone; the other groups received free ICG, UCNPs-I, or UCNPs-DI and then went through PW/CW corresponding irradiation (980 nm, 0.5 W/cm^2^ for 10 min). The changes in tumor volume and body weight were then monitored and recorded over the next 21 days. As shown in Fig. 6b, e, no obvious tumor growth inhibition effect was observed in the group with only UCNPs-DI treated during the whole treatment. By contrast, the tumor received the nanoagent and CW 980 nm laser treatment, the volume shrunk persistently, and the tumor growth was almost completely inhibited after treatment for 21 days, as depicted in Fig. 6c. Similar therapeutic trends were observed between the cohorts of free ICG and UCNPs-I. Figure 6d shows few changes in the body weight of the mice during feeding time, indicating that UCNPs-DI possesses high therapeutic efficiency and the systemic toxicity of the UCNPs-DI nanoagent with PW/CW irradiation was insignificant.

In order to further confirm the anti-tumor effect of UCNPs-DI, we used immunohistochemical staining for histological analysis to evaluate the pathological changes of the tumor tissue collected after the above treatment. The remarkable green SOSG fluorescent signal was observed only in the tumor treated with UCNPs-I and UCNPs-DI under the CW laser irradiation, respectively, further indicating that the photogenerated massive ^1^O_2_ was original from the activation of ICG by virtue of the upconverted optical energy (Fig. 6f). In addition, we also performed hematoxylin and eosin (H&E) staining assays to study tumor cell death and organ damage after the phototherapy on the 21st day (Fig. 6g). H&E-staining of the tumor slices indicated that the deformed nucleus (karyopyknosis, karyorrhexis, and karyolysis) appeared in the UCNPs-DI + CW laser group, demonstrating significant inhibition effect to the proliferation capability of tumor cells, which is consistent with the above-mentioned therapeutic results in vivo. The photographs of the pathomorphological analysis of heart, liver, spleen, lung, and kidney are provided in Supplementary Fig. 25, which shows no obvious organ damage in all cases, indicating the good biocompatibility in vivo of the as-prepared UCNPs-DI. Taken together, these results reveal that we can achieve the in vivo orthogonal safe PA imaging and highly effective phototherapy in the UCNPs-approved nanoagent through programmably switching the on-duty ratio of the excitation laser with the same wavelength.

## Discussion

In summary, we have successfully demonstrated a facile but efficient strategy to switch the NIR 800 nm emission of the UCNPs by programmable regulating the on-duty ratio of the excitation laser to achieve the orthogonal real-time safe photoacoustic imaging and effective switching on-demand photodynamic therapy with a rationally designed nanoagent. Our discovery provides an effective referential protocol to tune the upconvertion emission spectra by controlling the energy transfer between the sensitizer and the activators through simply adjusting the pulse width of the laser. The nanoagent UCNPs-DI is composed of a highly visible-absorbing polymer dye DPP and an NIR photosensitizer ICG loaded on the surface of the UCNPs. UCNPs-DI with programmably controlling upconversion process can efficiently transduce the 980 nm excitation to the steady-state visible light and dynamic NIR light. The spectral overlap between the absorption of DPP and the upconverted visible emission makes better use of the highly emissive upconversion to determine the lesion area through photoacoustic imaging under 980 nm pulsed laser irradiation, and in particular, no photothermal and photodynamic effects occur during the measurement. By switching the excitation light into CW mode to lighten the 800 nm emission, the ICG can be activated to generates cytotoxic ROS for antitumor therapy. In short, with UCNPs-DI, we can avoid the harmful effects caused by the ROS photooxidation and photohyperthermia during the long-term and real-time PA imaging diagnosis and achieve effective PA imaging-guided phototherapeutics. This work provides a facile approach for switchable NIR upconversion emission to achieve the orthogonal activation of imaging diagnostics and photodynamic therapeutics toward the target cancers.

## Methods

### Chemicals

The chemicals mentioned in this article were all reagent grade and were used without further purification. Rare earth acetates X(CH_3_COO)_3_·4H_2_O (X = Y, Yb, Er, Tm), 1-octadecane (ODE), oleic acid (OA), ammonium fluoride (NH_4_F), indocyanine green (ICG), 3-(4,5-Dimethylthiazol-2-yl)-2,5-diphenyltetrazolium bromide (MTT), calcein-acetylmethoxylate (calcein-AM), propidium iodide (PI) and 4-nitrophthalonitrile fluorescein isothiocyanate (FITC) were purchased from Sigma-Aldrich Corporation (MO, USA). 2,5-Bis(trimethylstannyl)thieno[3,2-b]thiophene (THP) and 3,6-Bis(5-bromothiophen-2-yl)-2,5-bis(2-decyltetradecyl)pyrrolo[3,4-c]pyrrole-1,4(2H,5H)-dione (DP) were purchased from Derthon Optoelectronic Materials Science & Technology Co., Ltd. (Shenzhen, China). 1,2-distearoyl-sn-glycero-3-phosphoethanolamine-N-[methoxy(polyethyleneglycol)-2000 (DSPE-PEG-COOH) was purchased from Avanti Polar Lipids Inc. (AL, USA). SOSG was obtained from Thermo Fisher Scientific Co, Ltd. (China). Fetal bovine serum (FBS), Dulbecco’s modified Eagle’s medium (DMEM), and penicillin were purchased from GIBCO. Deionized (DI) water comes from Millipore Milli-Q water purification system (Billerica, USA).

### Material characterization

The absorbance spectra of UCNPs, DPP, ICG, UCNPs-D, UCNPs-I, and UCNPs-DI under various conditions were measured by an ultraviolet/visible absorption spectrometer (Lambda-35 UV/visible spectrophotometer, Perkin-Elmer, MA, USA). Fluorescence spectra were determined using LS-55 fluorescence spectrophotometer (Perkin-Elmer, MA, USA). Upconversion luminescence spectra were obtained on the Ocean Insight spectrometer with Spetrasuite software (Florida, USA). The PA signal intensities were recorded by a 10 MHz, 10 mJ/cm^2^, 384-element ring ultrasound array, and an optical parametric oscillator (OPO) (BB-OPO-NIR, Deyang-Tech, Zhejiang, China; pump laser, Nimma-900, Beamtech, Beijing, China) with 5–10 ns pulse duration and 10 Hz pulse repetition rate was used as the light source. The TEM images of UCNPs and UCNPs-DI were captured by a high-resolution 2100F field emission transmission electron microscope (JEOL, Japan) operating at a capture acceleration voltage of 200 kV. ZEN3690 zeta sizer (Malvern, USA) was used to measure the particle size. The Bio-Rad FTS 6000 spectrometer (Bio-Rad Company, Hercules, California, USA) was used to record the FT-IR spectrum in the form of KBr particles. Flow cytometry assay was performed on CytoFLEX (Beckman Coulter, USA). Fluorescence imaging of the cells was acquired on the Olympus Fluoview FV 3000 microscope (Olympus Imaging America, Japan).

### Synthesis of UCNPs-DI

DSPE-PEG-COOH (2.8 mg, 1 μmol) was dispersed in 1 mL DI water, and next 400 μL of tetrahydrofuran solution, which contains DPP (0.4 mg/mL), UCNPs (2 mg/mL) and ICG (0.2 mg/mL), were added to the DSPE-PEG-COOH solution. The solution was sonicated for 10 min and stirred overnight at room temperature. After centrifuging, the crude product was scrubbed three times with DI water. Then the final nanoagent UCNPs-DI was obtained by dialyzed against PBS buffer (pH 7.4) using a cellulose membrane for 72 h. Then UCNPs-DI was redispersed in PBS solution and deposited in 4 °C for use. UCNPs-D and UCNPs-I was obtained by similar assembly procedures to encapsulate only DPP dye and ICG dye on the surface of UCNPs, respectively.

### Detection of singlet oxygen production

SOSG was used to detect the ^1^O_2_ generation of UCNPs-DI under irradiation of 980 nm PW (10 ns)/CW laser or ICG under irradiation of 980/808 nm CW laser. SOSG dissolved in ethanol (33 μL) was added into 3 mL PBS. Then, the SOSG solution (100 μL) was appended to 1 mL of UCNPs-DI (200 μg/mL in PBS) or ICG (10 μg/mL in DI water) solution. The solution was irradiated under laser radiation (0.5 W/cm^2^) with different time. The fluorescence spectra of these solutions were record (fluorescence determined by using 488 nm excitation).

### Cell culture and photocytotoxicity testing

MCF7 (Human breast adenocarcinoma cells), HepG2 (Human hepatocellular carcinoma cells), and EMT6 (Murine breast cancer cells) cells were obtained from the American Type Culture Collection (ATCC) and cultured in 1% and penicillin/streptomycin DMEM containing 10% fetal bovine serum after gamma irradiation and sterile filtration. The culture environment of the cells is a humid environment with a temperature of 37 °C and a CO_2_ concentration of 5%.

MCF7 cells were planted into a 96-well plate and cultured in 5% CO_2_ at 37 °C for 24 h. After UCNPs-DI at concentrations of 0, 25, 50, 100, 150, and 200 μg/mL was added to the wells, respectively, and incubation for 4 h, the medium was removed from wells and replaced with fresh medium. Then the 96-well plate was irradiated under 980 nm PW laser (10 ns, 0.5 W/cm^2^) or CW laser (0.5 W/cm^2^) for 3 min. At 24 h post-laser irradiation, 10 μL MTT was added to the 96-well plate for an additional 4 h incubation. 100 μL sodium dodecyl sulfate (SDS) was subsequently added for another 4 h of incubation. We used a microplate reader to measure and record the absorbance of MTT (Infinite 200, TECAN, Switzerland). Finally, cell viability is determined by the following equation: cell viability (%) = (average absolute value of the treatment group/average absolute value of the control) × 100. Dead/live cell co-staining was also performed under similar procedures, in which at 12 h post-laser irradiation, each plate was incubated with 1 mL of dye solution (2 μM calcein AM and 4 μM PI), co-stained for 30 min at 37 °C, and imaged using CLSM. The phototoxicity of UCNPs-DI (200 μg/mL) under irradiation of laser with different power density to MCF7 cells and the phototoxicity of UCNPs-DI (200 μg/mL) under excitation of 980 nm PW laser (10 ns, 0.5 W/cm^2^) and CW laser (0.5 W/cm^2^) to HepG2 and EMT6 cells were performed with similar experimental procedures.

Flow cytometry analysis: MCF7 cells were seeded in six-well plates and cultured for 24 h until all cells were fully attached. The cells were treated with UCNPs-DI (200 μg/mL) for 4 h and recultured in fresh medium. Then the plate was irradiated under 980 nm PW laser (10 ns, 0.5 W/cm^2^) or CW laser (0.5 W/cm^2^) for 3 min. 24 h later, all cells were harvested and collected in EP tubes, and washed twice (2000 rpm, 3 min, 4 °C) with cold PBS, and then suspended in 100 µL binding buffer. Then, 5 µL Annexin V-FITC (20 µg/mL) was added to each tube and incubated for 15 min at 4 °C in dark. After gentle vortex, 2 µL PI (50 µg/mL) was added to each tube. The cells were filtered with Falcon™ Cell Strainers prior to the flow cytometry analysis. The cells without any stain were set as negative control.

### Intracellular singlet oxygen detection

MCF7 cells were seeded in a 35 mm confocal culture dish and cultured for 24 h. The cells were treated with UCNPs-DI (200 μg/mL) for 4 h and then treated with SOSG (5 μM, 50 μL in PBS) for 1 h in the dark. After replaced with fresh medium, the plate was irradiated under 980 nm PW laser (10 ns, 0.5 W/cm^2^) or CW laser (0.5 W/cm^2^) for 3 min. Fluorescence imaging was then recorded at 500–550 nm (the excitation laser for SOSG is 488 nm, and the fluorescence emission is 526 nm) by CLSM.

### Tumor mouse model

Female BALB/c mice (~4 weeks) were purchased from the Animal Experiment Center of Southern Medical University and raised in a sterile mouse house. The mice were maintained with free access to food and water in an environment of ambient temperature (~23 ± 3 °C), 40–70% humidity, and 12 h light/dark cycles. All animal procedures comply with the Guidelines for the Care and Use of Laboratory Animals issued by the National Institutes of Health (NIH) of South China Normal University, and the experiments have been approved by the Animal Ethics Committee of South China Normal University. The maximum permitted tumor volume (2000 mm^3^) was not exceeded in any study.

The EMT6 tumor cells were transferred and evenly distributed in 100 μL PBS, and injected subcutaneously into the back of each mouse. When the tumor reached about 100 mm^3^, the animals were tested. The calculation formula of tumor volume is: tumor volume = length (maximum longitudinal diameter) × width (maximum transverse diameter)^2^ × 0.5.

### Photoacoustic imaging in tumor in vivo

BALB/c mice bearing EMT6 tumors were anesthetized and then injected with 100 μL UCNPs-DI (200 μg/mL) or free ICG (1.0 mg/mL) through the tail vein. Then, at different time intervals post-injection, the tumor region was imaged using the photoacoustic computed tomography system equipped with a 10 MHz, 10 mJ/cm^2^, 384-element ring ultrasound array, and an optical parametric oscillator (OPO) (BB-OPO-NIR, Deyang-Tech, Zhejiang, China; pump laser, Nimma-900, Beamtech, Beijing, China) with 5–10 ns pulse duration and 10 Hz pulse repetition rate was used as the light source. A LabVIEW program was used to control the photoacoustic imaging system and acquire images. The photoacoustic images were processed with Matlab software. The PA imaging of DPP (400 μg/mL, encapsulated in DSPE-PEG-COOH), UCNPs-D (200 μg/mL) and UCNPs-I (200 μg/mL) was captured at post-injection 12 h, respectively. Mice were anaesthetized using 4% chloral hydrate through intraperitoneal injection before imaging.

### In vivo tumor phototherapy

BALB/c mice bearing EMT6 tumors were divided into 10 groups randomly (6 mice per group, every phototreatment mouse bearing two subcutaneous tumors on the left and right sides received the same sample injection but different PW/CW irradiations on the two tumors). Among, PBS: blank group injected with PBS only; PW: control group only irradiated with 980 nm pulsed laser (10 ns, 0.5 W/cm^2^, 10 min); CW: control group only irradiated with 980 nm continuous laser (0.5 W/cm^2^, 10 min); UCNPs-DI: control group only injected intravenously with 100 μL UCNPs-DI (200 μg/mL); PW + ICG: control group injected intravenously with 100 μL ICG (200 μg/mL) and irradiated with 980 nm pulsed laser (10 ns, 0.5 W/cm^2^, 10 min); CW + ICG: control group injected intravenously with 100 μL ICG (200 μg/mL) and irradiated with 980 nm continuous laser (0.5 W/cm^2^, 10 min); PW + UCNPs-I: control group injected intravenously with 100 μL UCNPs-I (200 μg/mL) and irradiated with 980 nm pulsed laser (10 ns, 0.5 W/cm^2^, 10 min); CW + UCNPs-I: control group injected intravenously with 100 μL UCNPs-I (200 μg/mL) and irradiated with 980 nm continuous laser (0.5 W/cm^2^, 10 min); PW + UCNPs-DI: control group injected intravenously with 100 μL UCNPs-DI (200 μg/mL) and irradiated with 980 nm pulsed laser (10 ns, 0.5 W/cm^2^, 10 min); CW + UCNPs-DI: treatment group injected intravenously with 100 μL UCNPs-DI (200 μg/mL) and irradiated with 980 nm continuous laser (0.5 W/cm^2^, 10 min). The 10 groups of mice were injected with samples through the tail vein every 2 days, and the tumor area was treated with laser at post-injection 12 h. During the treatment, the mice were anesthetized with 4% chloral hydrate. The treatment effects of the 10 cohorts were appraised by detecting and recording the relative tumor volume and weight changes of mice. Finally, the tumors were extracted at 21 d post-irradiation at the end of this experiment.

### Detection of singlet oxygen in vivo

BALB/c mice bearing with EMT6 tumors were intravenously injected with 100 μL UCNPs-DI (200 μg/mL) or other controls. At the post-injection 12 h, 30 μL SOSG (50 μM) was injected into the tumor tissue of the mouse. Then the tumor region was irradiated under 980 nm PW laser (10 ns, 0.5 W/cm^2^) or CW laser (0.5 W/cm^2^) for 10 min. After the mice were euthanized, the tumor tissues of the mice were dissected, and the tumor tissues were fixed in 4% paraformaldehyde and then cryosectioned into sections at a thickness of 10 μm. The tumor sections were stained with DAPI (4, 6-diamidino-2-phenylindole) and then observed under the CLSM.

### Histology examination

After the treatment, the mice were euthanized. The tumor tissues and major organs (heart, liver, spleen, lung and kidney) were removed from 10 groups of mice and cut into sections with thickness of 4 μm. The tumor sections were fixed in 4% paraformaldehyde solution for 12 h, then dehydrated with ethanol and processed into paraffin. Finally, the tissue section was stained with H&E and recorded and imaged by a fluorescence microscope system (Nikon E 200). The H&E staining method is processed according to the method provided by the supplier (BBC Biochemical).

### Statistics and reproducibility

The TEM images of UCNPs and UCNPs-DI in Fig. 3b inset and Supplementary Fig. 1a, b were repeated thrice independently with similar results, and one representative image from each group was shown. Confocal imaging of MCF7 cells incubated with or without UCNPs-DI at PW or CW irradiation were independently repeated at least three times with similar results, and a series of representative images from each group were shown in Fig. 4c, d. In vivo PA imaging of mice at post-injection of UCNPs, DPP, ICG, UCNPs-D, UCNPs-I, and UCNPs-DI was independently repeated at least twice using biologically independent mice with similar results, and a series of representative images were shown, such as Fig. 5b, c. The evaluation of in vivo phototherapeutic effects of PW, CW, UCNPs-DI, PW + ICG, CW + ICG, PW + UCNPs-I, CW + UCNPs-I, PW + UCNPs-DI, and CW + UCNPs-DI via SOSG and H&E staining was independently repeated at least twice using biologically independent mice with similar results, and a series of representative images were shown in Fig. 6f, g and Supplementary Fig. 25.

### Statistical analysis

Quantitative data are expressed as mean ± SD. *P* values were calculated by two-sided Student’s t-test. All statistical analyses were performed using GraphPad Prism Software (Version 9.0) or Origin Software (Version 8.0).

### Reporting summary

Further information on research design is available in the Nature Research Reporting Summary linked to this article.

## Supplementary information

Supplementary Information

Reporting Summary

## Data Availability

The data supporting the findings in this study are available within the manuscript, its Supplementary Information file and the Source Data file. Source data are provided with this paper.

## Source data

Source Data
