# Supplementary material for: Switching the NIR upconversion of nanoparticles for the orthogonal activation of photoacoustic imaging and phototherapy
Source: Nat Commun. 2022 Jun 7;13:3149. doi: 10.1038/s41467-022-30713-w (PMC9174188; doi:10.1038/s41467-022-30713-w)
Supplement: Supplementary file 1 — Supplementary Information [file 41467_2022_30713_MOESM1_ESM.pdf]

Supplementary Information for

Switching the NIR upconversion of nanoparticles for the orthogonal activation of photoacoustic imaging and phototherapy

Yang Yang,[1] Jinshu Huang,[2] Wei Wei,[1] Qin Zeng,[1] Xipeng Li,[1] Da Xing,[1] Bo Zhou,[2]* Tao Zhang[1,3]*

[1]MOE Key Laboratory of Laser Life Science & Institute of Laser Life Science, Guangdong Provincial Key Laboratory of Laser Life Science, College of Biophotonics, South China Normal University, Guangzhou 510631, China

[2]State Key Laboratory of Luminescent Materials and Devices, South China University of Technology, Guangzhou 510641, China

[3]Guangzhou Key Laboratory of Spectral Analysis & Functional Probes, College of Biophotonics, South China Normal University, Guangzhou 510631, China

Email: zhoubo@scut.edu.cn (B. Zhou); zt@scnu.edu.cn (T. Zhang)

Table of content of

Supplementary Information

1	Supplementary Methods	Page 3
2	Supplementary Figs. 1-3	Page 4
3	Supplementary Figs. 4-6	Page 5
4	Supplementary Figs. 7-8	Page 6
5	Supplementary Figs. 9-11	Page 7
6	Supplementary Figs. 12-14	Page 8
7	Supplementary Figs. 15-17	Page 9
7	Supplementary Fig. 18	Page 10
5	Supplementary Figs. 19-20	Page 11
6	Supplementary Figs. 21-23	Page 12
7	Supplementary Figs. 24-26	Page 13

Supplementary Methods

Synthesis of UCNPs

UCNPs were prepared by an improved solvothermal method. Generally, $Er(CH_3COO)_3{\bullet}4H_2O$ (0.015 mmol), $Tm(CH_3COO)_3{\bullet}4H_2O$ (0.003 mmol), $Yb(CH_3COO)_3{\bullet}4H_2O$ (0.20 mmol), $Y(CH_3COO)_3{\bullet}4H_2O$ (0.782 mmol), OA (6 mL) and ODE (15 mL) were mixed into a 100 mL flask. The solution temperature was increased to 120 °C, at which the solution was stirred for 30 min and then temperature was decreased to 37 °C. Then, a solution of NH_4F (4 mmol) and NaOH (2.5 mmol) dissolved in 10 mL of methanol was tardily added to the flask. Thereafter, the solution was heated to 50 °C for 30 min and further heated to 120 °C for another 30 min to remove the methanol with degassing, and then heated under nitrogen to 300 °C for 90 min. At last, the solution was allowed to cool, precipitated with ethanol from the solution of nanocrystal, collected by centrifugation (6000 r, 3 min) and then washed with cyclohexane/ethanol (1:1, v/v) for 3 times to afford the product UCNPs.

Synthesis of DPP

The DPP polymers was synthesized by Stille cross-coupling of the donor and acceptor moiety. 2,5-Bis(trimethylstannyl)thieno[3,2-b]thiophene (THP, 116 mg, 0.25 mmol), 3,6-Bis(5-bromothiophen-2-yl)-2,5-bis(2-decyltetradecyl)pyrrolo[3,4-c]pyrrole-1,4(2H,5H)-dione (DP, 283 mg, 0.25 mmol), and palladium catalyst $Pd(PPh_3)_4$ (5 mg, 0.004 mmol) were stirred together and dissolved in 10 mL toluene. Then, the mixture was degassed and filled with nitrogen through three freeze-pump-thaw cycles. Under the protection of sealed nitrogen, the reaction was stirred at 100 °C for 24 hours. Then 20 mg pinacol phenylboronate and 0.2 mL bromobenzene was added to remove the reactants. After the solution was cooled to room temperature, methanol was added and the precipitate was gathered by filter paper. The polymer is purified by Soxhlet extraction using a mixture of methanol and acetone to afford the crude product. The crude product was dissolved chloroform and filtration using a 0.22-μm membrane filter, and the filtrate was precipitated with 100 mL methanol to afford the product DPP.

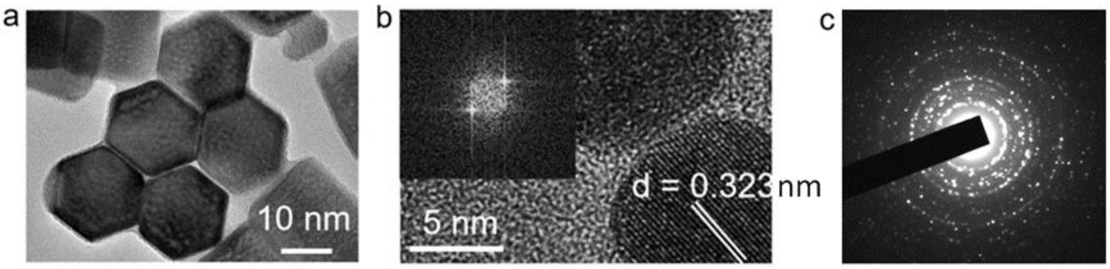

Supplementary Fig. 1. Characterization of UCNPs particles. (a) TEM images of UCNPs. (b) HRTEM micrograph of the UCNPs. Inset, FFT image. (c) The selected area electron diffraction (SAED) pattern of UCNPs.

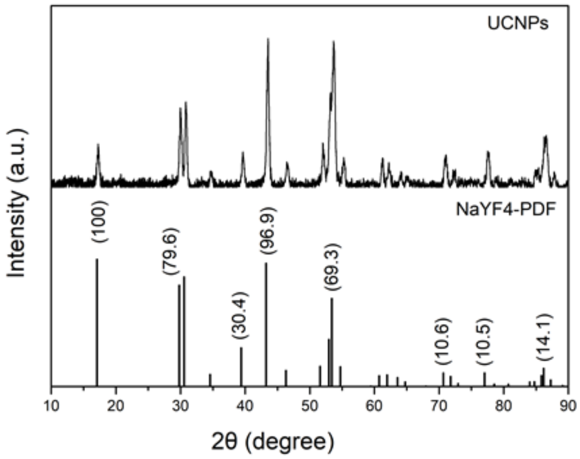

Supplementary Fig. 2. XRD spectrum of the UCNPs. As a reference, the pattern corresponding to the $NaYF_4$ (PDF#83-0074) is also shown.

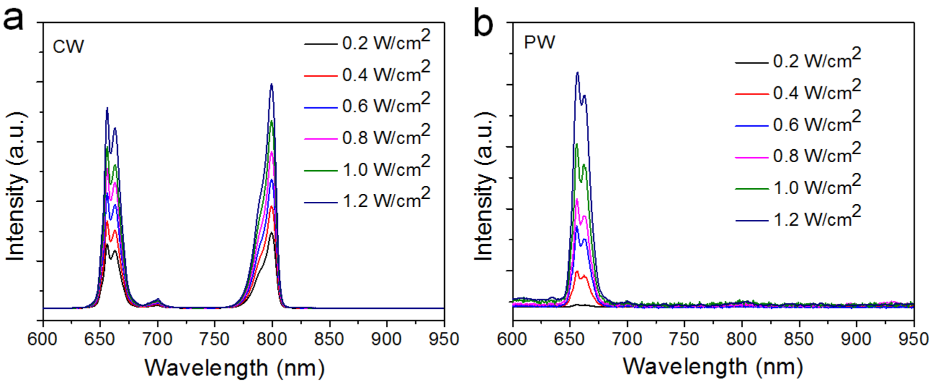

Supplementary Fig. 3. Upconversion emission spectra of the UCNPs under (a) CW and (b) PW 980 nm excitations at different pump power densities.

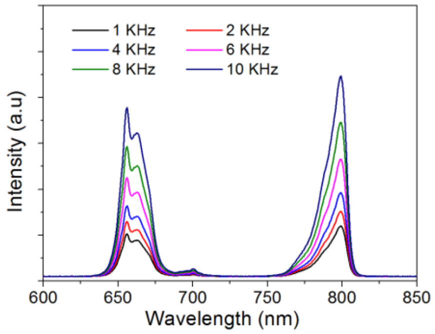

Supplementary Fig. 4. Upconversion emission spectra of UCNPs-DI under 980 nm PW excitation with different frequency.

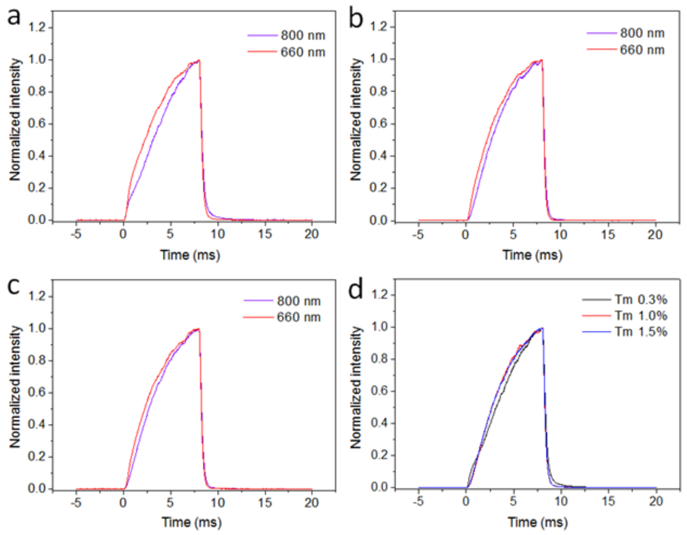

Supplementary Fig. 5. Time-dependent upconversion emission profiles of Er^{3+} at 660 nm and Tm^{3+} at 800 nm for the $NaYF_4$:20%Yb,1.5%Er,0.3-1.5%Tm UCNPs with Tm^{3+} concentrations of (a) 0.3%, (b) 1.0% and (c) 1.5% under pulse 980 nm excitation. (d) A comparison of the time-dependent upconversion emission profiles of Tm^{3+} at 800 nm from the (a-c) samples.

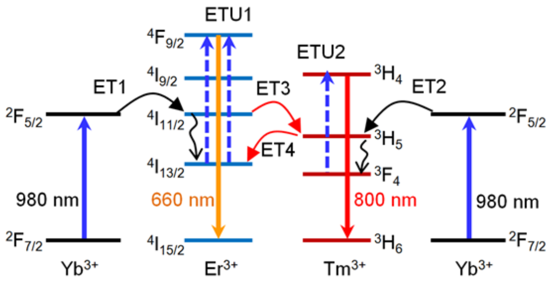

Supplementary Fig. 6. Schematic of promoting the red upconversion of Er^{3+} through the energy circling of Er^{3+} ($^4I_{11/2}$) → Tm^{3+} ($^3H_5$) → Er^{3+} ($^4I_{11/2}$). TE3: Er^{3+} ($^4I_{11/2}$) → Tm^{3+} ($^3H_5$); ET4: Tm^{3+} ($^3H_5$) → Er^{3+} ($^4I_{13/2}$). The meaning of other marks can refer to Fig. 2d in main text.

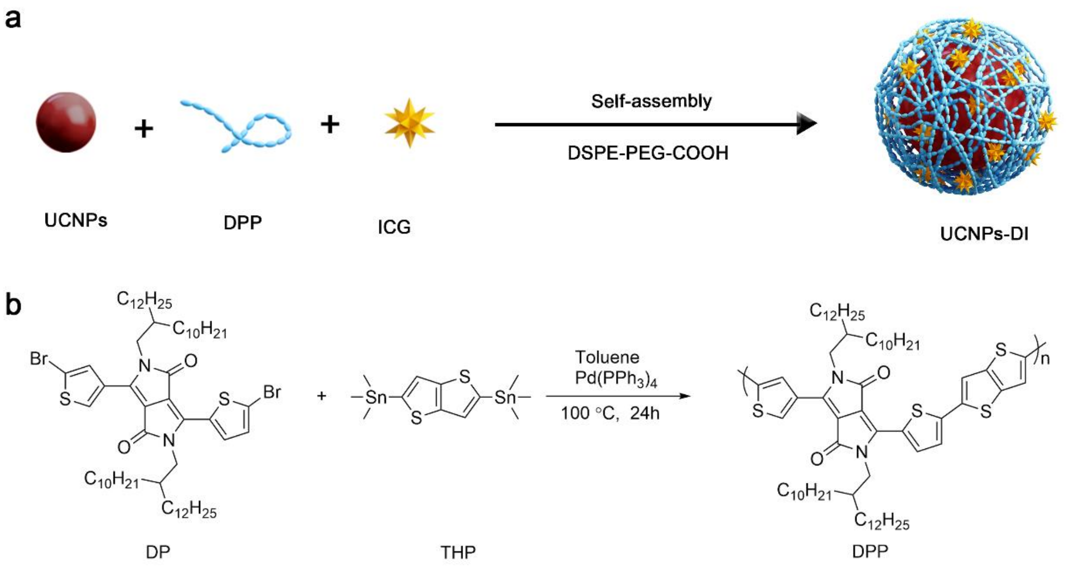

Supplementary Fig. 7. Synthestic route of (a) UCNPs-DI and (b) DPP.

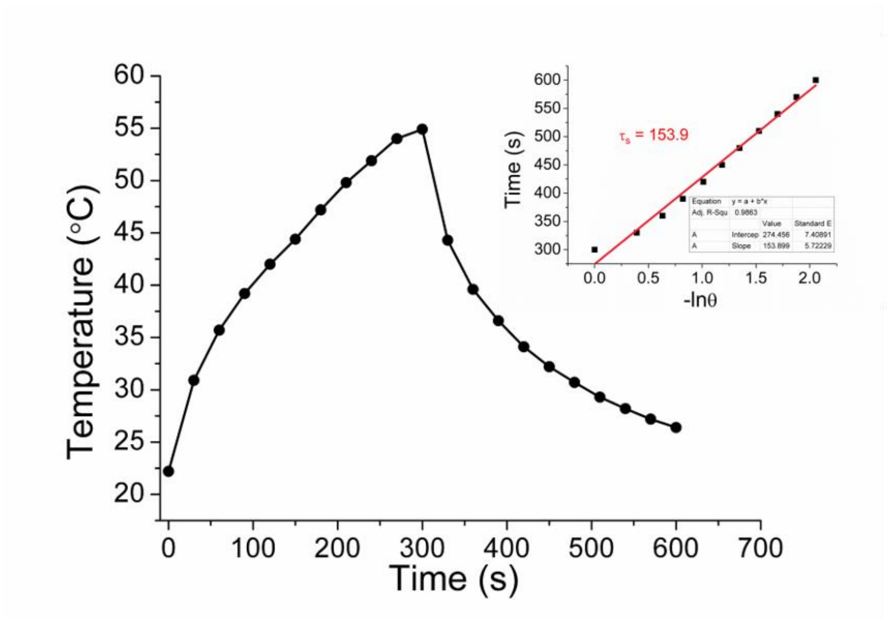

Supplementary Fig. 8. Photothermal properties of DPP. Plots of the temperature *vs* time for the DPP. Insert, the plots of cooling time *vs* -lnθ. On the basis of the linear regression analysis, the time constant for heat transfer τ_s (the slope of the plot) was determined to be 153.90 s.

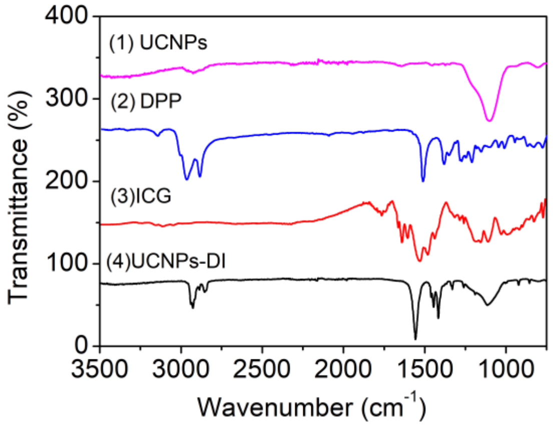

Supplementary Fig. 9. FTIR transmission spectra of UCNPs, DPP, free ICG and UCNPs-DI.

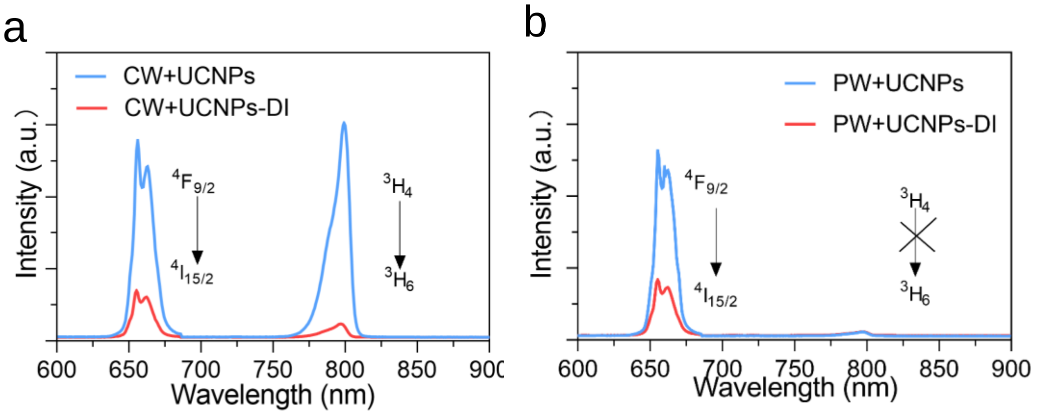

Supplementary Fig. 10. Upconversion luminescence spectra of UCNPs and UCNPs-DI under excitation of (a) 980 nm CW laser and (b) 980 nm PW laser (10 ns).

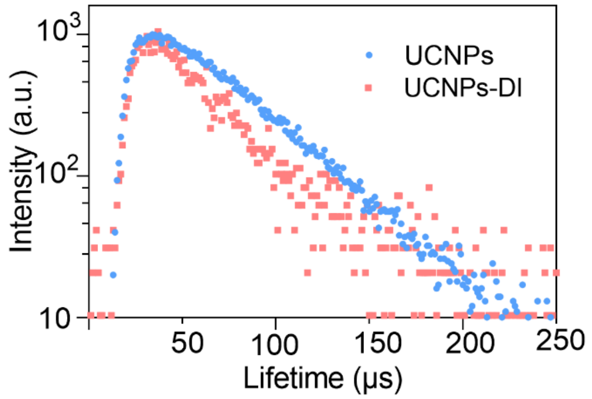

Supplementary Fig. 11. Luminescence decay curves of the emission at 800 nm for UCNPs and UCNPs-DI excited by 980 nm laser (0.5 W/cm^2).

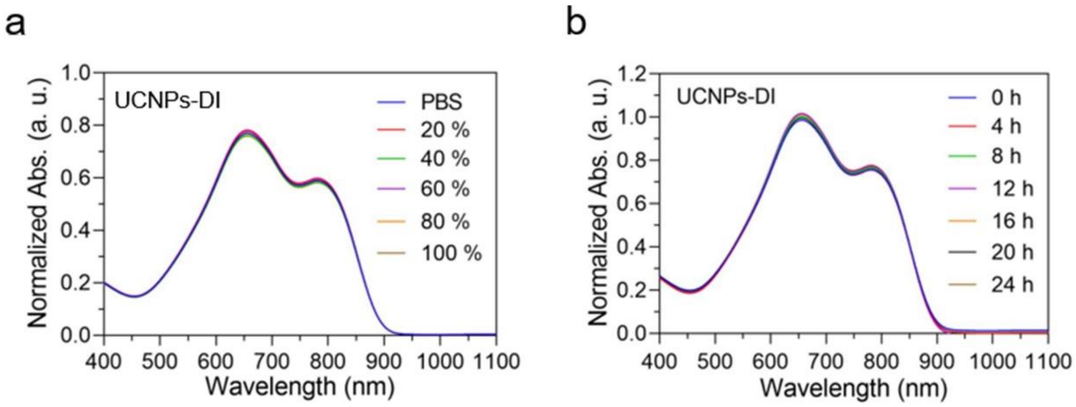

Supplementary Fig. 12. Stability of UCNPs-DI. Absorption spectra of UCNPs-DI in PBS (pH = 7.4) with (a) different content of serum (filtrate, from 0 to 100%) and (b) 100% serum keeping for various time (from 0 to 24 h).

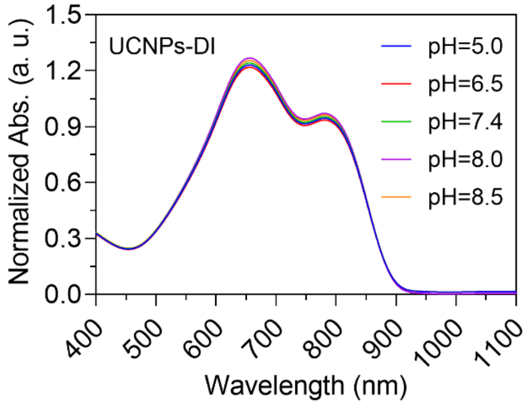

Supplementary Fig. 13. Stability of UCNPs-DI in different pH. Absorption spectra of UCNPs-DI in PBS with different physiological pH (from 5.0 to 8.5).

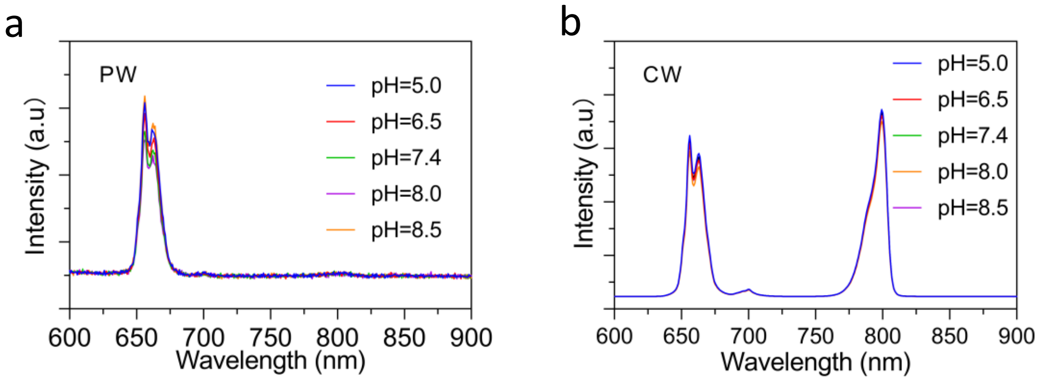

Supplementary Fig. 14. Changes in upconversion luminescence spectra of UCNPs-DI under excitation of (a) 980 nm PW laser and (b) 980 nm CW laser.

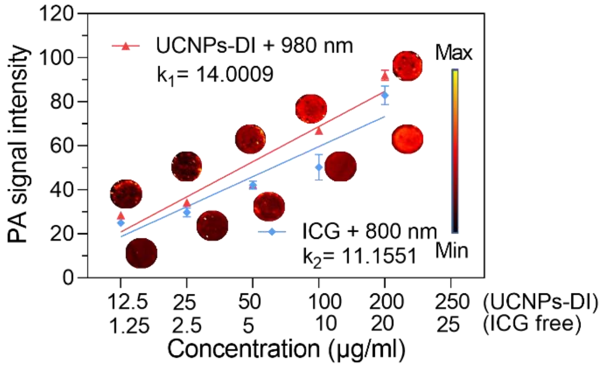

Supplementary Fig. 15. Comparison of the PA effect of UCNPs-DI and ICG. PA signal intensity of UCNPs-DI and ICG at different concentrations under excitation of 980 nm and 800 nm PW laser, respectively. Mean values and error bars are defined as mean and SD, respectively. All data are presented as mean ± SD (n = 3)

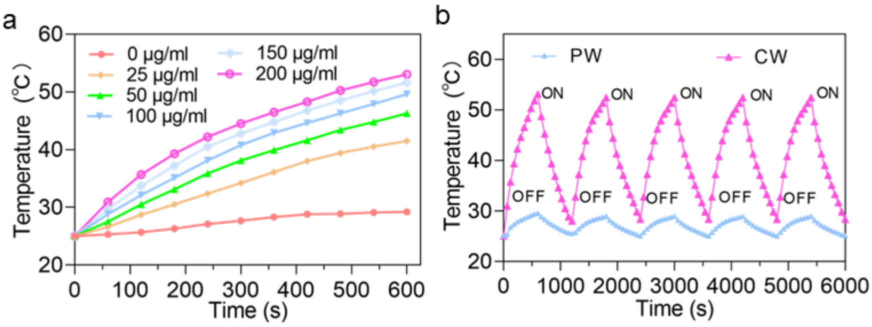

Supplementary Fig. 16. Photothermal behaviors of UCNPs-DI under PW or CW irradiation. (a) The photothermal heating curves of UCNPs-DI with different concentrations (0, 25, 50, 100, 150 and 200 μg/mL) under irradiation of 980 nm CW laser at the power density of 0.5 W/cm^2. (b) Temperature variations of the UCNPs and UCNPs-DI under the irradiation of 980 nm PW/CW laser at a power density of 0.5W/cm^2 for five light on/off cycles.

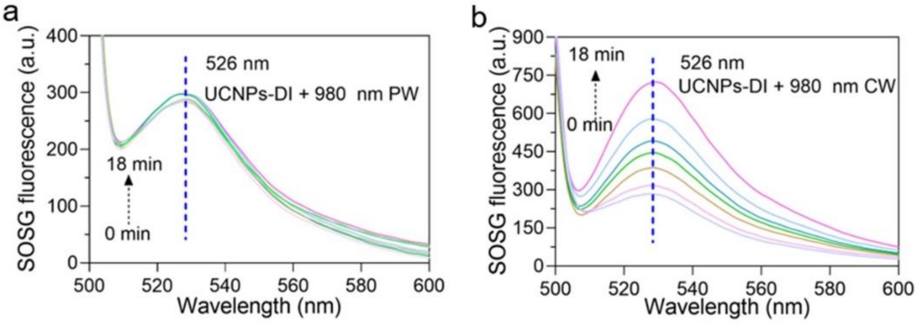

Supplementary Fig. 17. Singlet oxygen generation of UCNPs-DI under the irradiation of (a) 980 nm PW laser and (b) 980 nm CW laser.

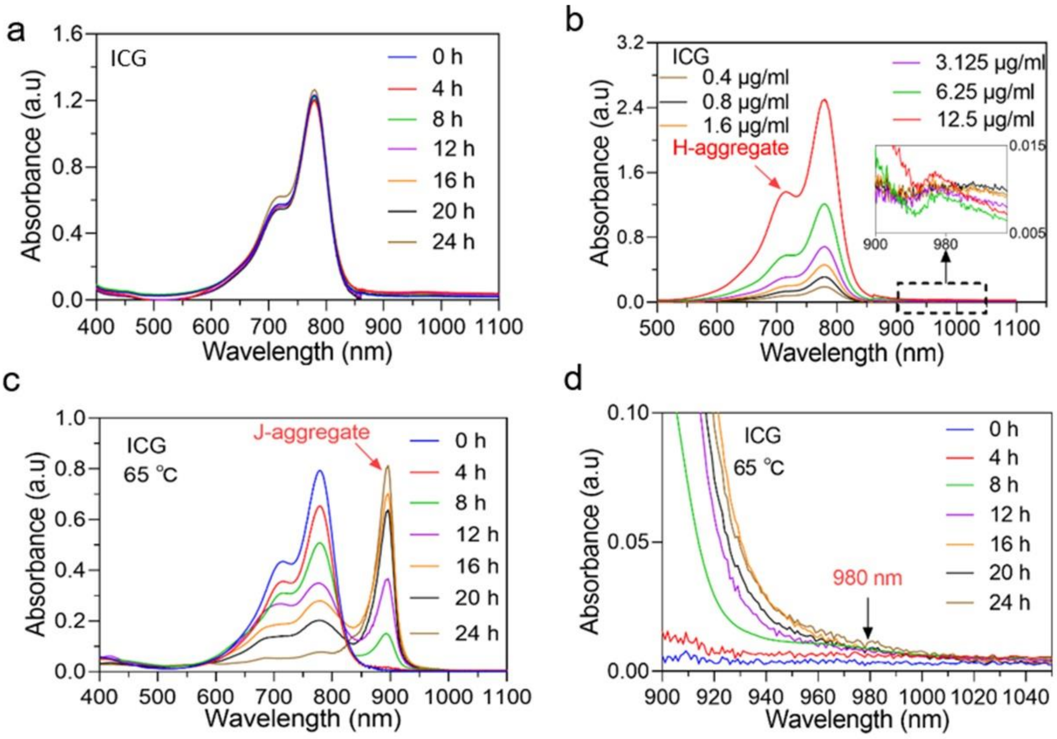

Supplementary Fig. 18. Absorption of ICG under different conditions. (a) Absorption spectra of ICG in D. -I. water with different standing time (fresh, 0 h to 24 h). (b) Absorption spectra of ICG at different concentrations. Inset, the enlarged absorption from 900 to 1100 nm. (c) Absorption spectra of ICG with different heating time at 65 °C. (d) Enlarged absorption spectra of (c) from 900 to 1050 nm, showing that the absorption of ICG at 980 nm was negligible and did not increase along with J-aggregates increase.

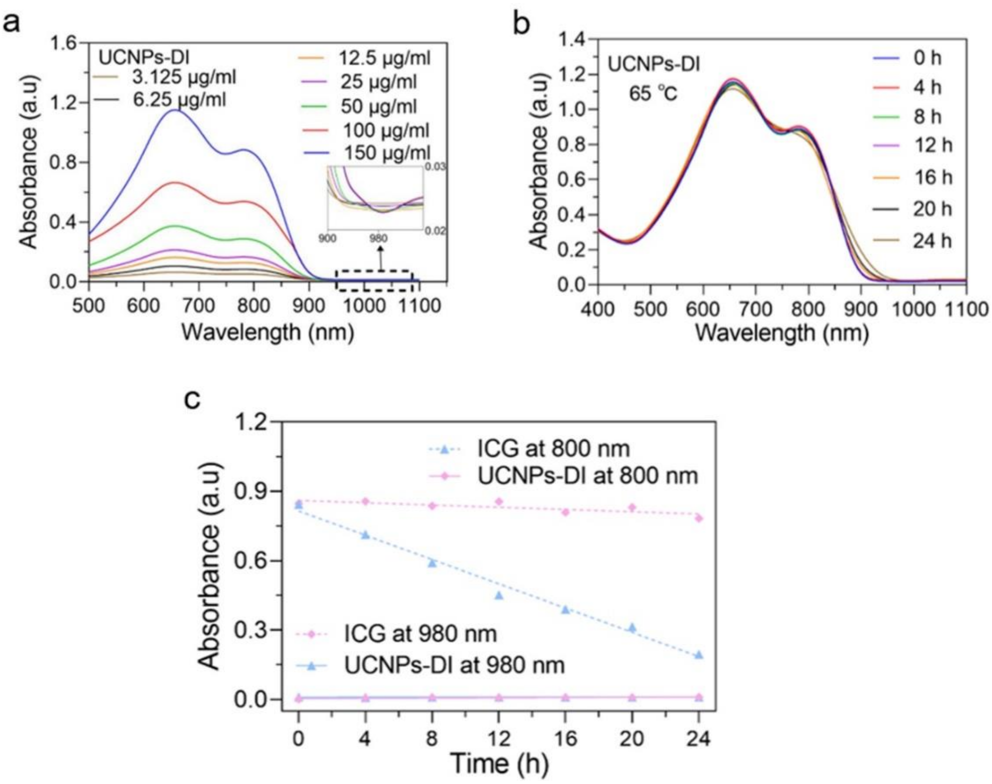

Supplementary Fig. 19. Absorption of UCNPs-DI under different conditions. (a) Absorption spectra of UCNPs-DI at different concentrations. Inset, the enlarged absorption from 900 nm to 1100 nm. (b) Absorption spectra of UCNPs-DI at different heating time at 65 °C. (c) A comparison of the absorbance of ICG and UCNPs-DI at 800 and 980 nm with different heating time at 65 °C, respectively.

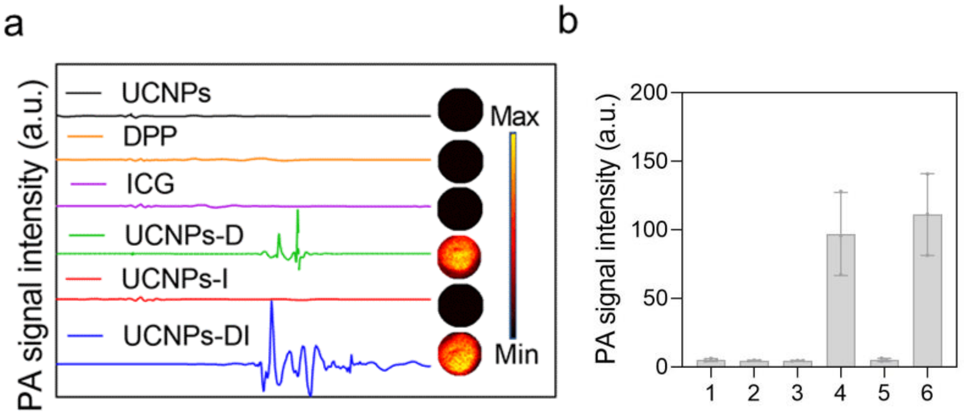

Supplementary Fig. 20. PA effects of UCNPs-DI and the controls. (a, b) PA signal intensity of UCNPs (1), DPP (2), ICG (3), UCNPs-D (4), UCNPs-I (5) and UCNPs-DI (6) under the excitation of 980 nm pulsed laser, respectively. Data are presented as mean ± SD (n = 3).

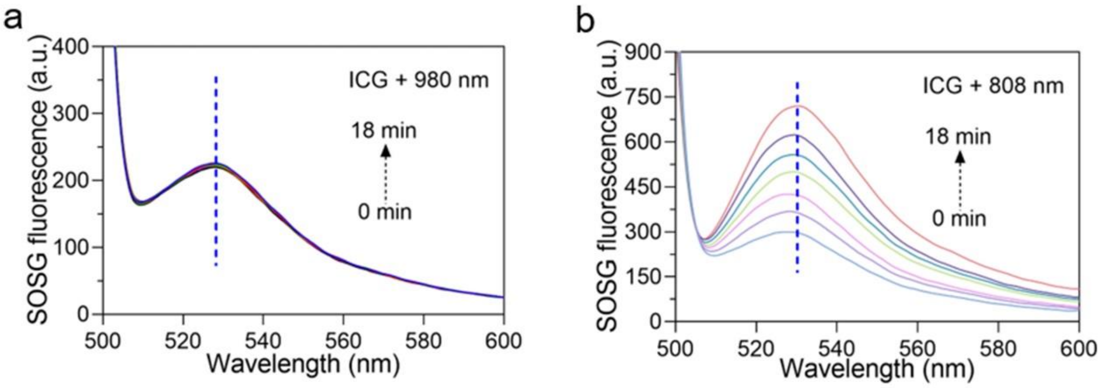

Supplementary Fig. 21. Singlet oxygen generation of free ICG under the irradiation of (a) 980 nm CW laser and (b) 808 nm CW laser.

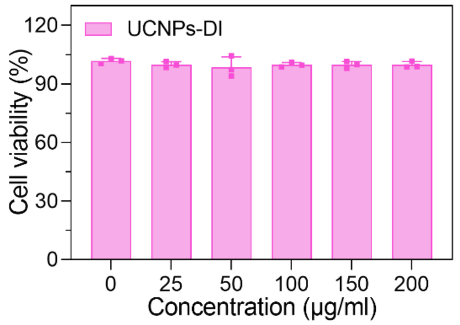

Supplementary Fig. 22. MCF7 cells were incubated with different concentrations of UCNPs-DI for 24 h, then the relative viabilities of MCF7 cells were determined by MTT assay. All data are presented as mean ± SD (n = 3).

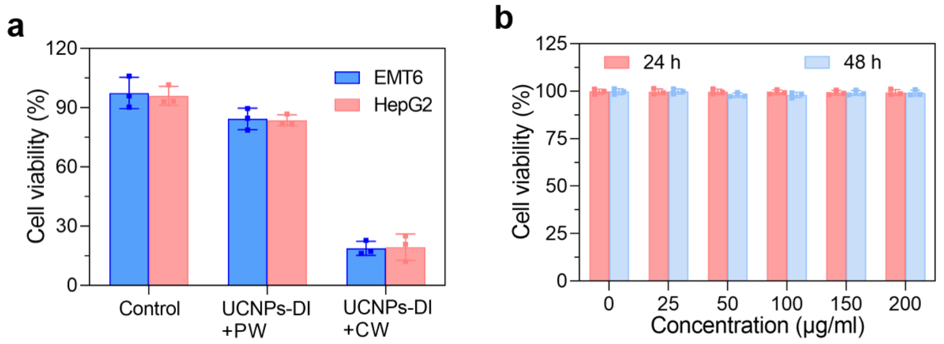

Supplementary Fig. 23. (a) Relative viabilities of EMT6 cells and HepG2 cells incubated with UCNPs-DI (200 μg/mL, 4h) under the irradiation of 980 nm PW (10 ns, 0.5 W/cm^2) or CW (0.5 W/cm^2) for 3 minutes. (b) LO2 cells were incubated with different concentrations of UCNPs-DI for 24 h or 48 h, then the relative viabilities of LO2 cells were determined by MTT assay. All data are presented as mean ± SD (n = 3).

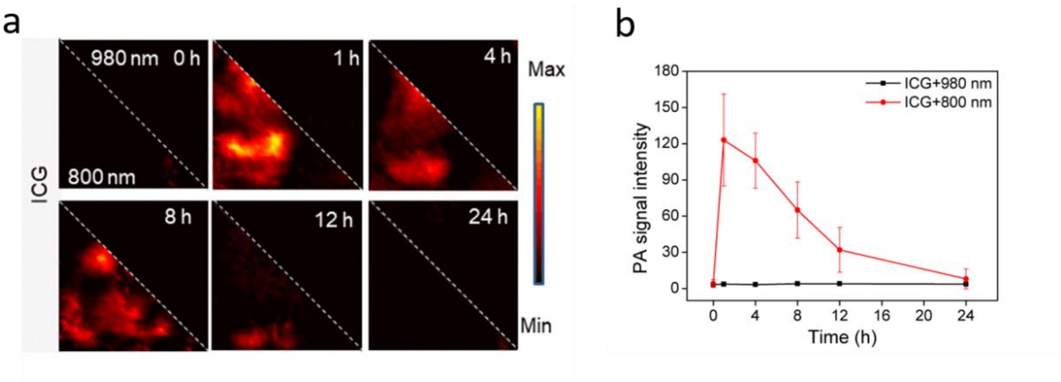

Supplementary Fig. 24. In vivo PA imaging of ICG. (a) Real-time photoacoustic images of the tumors after systematic administration of ICG in the mice under the excitation of 980 or 800 nm pulsed laser. (b) Quantitation of photoacoustic signals at the tumor sites of ICG. All data are presented as mean ± SD (n = 3).

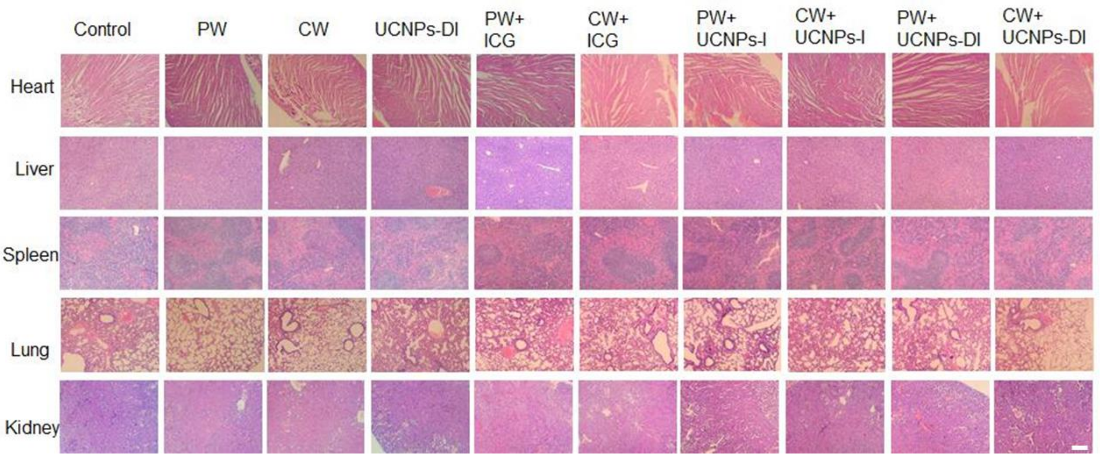

Supplementary Fig. 25. H&E-stained images of major organs from different treated mice. Scale bar, 100 µm.

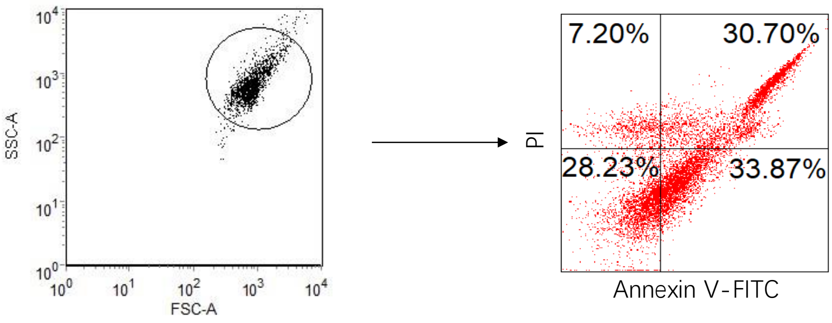

Supplementary Fig. 26. FACS gating strategies for apoptosis analysis (related to Figure 4e).
