# Supplementary material for: Switching the NIR upconversion of nanoparticles for the orthogonal activation of photoacoustic imaging and phototherapy
Source: Nat Commun. 2022 Jun 7;13:3149. doi: 10.1038/s41467-022-30713-w (PMC9174188; doi:10.1038/s41467-022-30713-w)
Supplement: Supplementary file 2 — Reporting Summary [file 41467_2022_30713_MOESM2_ESM.pdf]

nature portfolio

Corresponding author(s): Tao Zhang

Last updated by author(s): April 20, 2022

Reporting Summary

Nature Portfolio wishes to improve the reproducibility of the work that we publish. This form provides structure for consistency and transparency in reporting. For further information on Nature Portfolio policies, see our Editorial Policies and the Editorial Policy Checklist.

Statistics

For all statistical analyses, confirm that the following items are present in the figure legend, table legend, main text, or Methods section.

n/a	Confirmed	
☐	☒	The exact sample size (n) for each experimental group/condition, given as a discrete number and unit of measurement
☐	☒	A statement on whether measurements were taken from distinct samples or whether the same sample was measured repeatedly
☐	☒	The statistical test(s) used AND whether they are one- or two-sided<br>*Only common tests should be described solely by name; describe more complex techniques in the Methods section.*
☒	☐	A description of all covariates tested
☐	☒	A description of any assumptions or corrections, such as tests of normality and adjustment for multiple comparisons
☐	☒	A full description of the statistical parameters including central tendency (e.g. means) or other basic estimates (e.g. regression coefficient) AND variation (e.g. standard deviation) or associated estimates of uncertainty (e.g. confidence intervals)
☐	☒	For null hypothesis testing, the test statistic (e.g. F, t, r) with confidence intervals, effect sizes, degrees of freedom and P value noted<br>*Give P values as exact values whenever suitable.*
☒	☐	For Bayesian analysis, information on the choice of priors and Markov chain Monte Carlo settings
☒	☐	For hierarchical and complex designs, identification of the appropriate level for tests and full reporting of outcomes
☒	☐	Estimates of effect sizes (e.g. Cohen's d, Pearson's r), indicating how they were calculated

Our web collection on statistics for biologists contains articles on many of the points above.

Software and code

Policy information about availability of computer code

Data collection	Upconversion luminescence spectra were recorded on the Spetrasuite software. A LabVIEW program was used to control the photoacoustic imaging system and acquire images.
Data analysis	Statistical analyses were performed using Originlab 8.0, Microsoft Excel 2016 and ImageJ 1.52. Photoacoustic images were processed with Matlab software.

For manuscripts utilizing custom algorithms or software that are central to the research but not yet described in published literature, software must be made available to editors and reviewers. We strongly encourage code deposition in a community repository (e.g. GitHub). See the Nature Portfolio guidelines for submitting code & software for further information.

Data

Policy information about availability of data

All manuscripts must include a data availability statement. This statement should provide the following information, where applicable:

- Accession codes, unique identifiers, or web links for publicly available datasets
- A description of any restrictions on data availability
- For clinical datasets or third party data, please ensure that the statement adheres to our policy

The data supporting the findings in this study are available within the manuscript, its Supplementary Information file and the Source Data file. Source data are provided with this paper.

Field-specific reporting

Please select the one below that is the best fit for your research. If you are not sure, read the appropriate sections before making your selection.

☒ Life sciences ☐ Behavioural & social sciences ☐ Ecological, evolutionary & environmental sciences

For a reference copy of the document with all sections, see nature.com/documents/nr-reporting-summary-flat.pdf

Life sciences study design

All studies must disclose on these points even when the disclosure is negative.

Sample size	No sample size calculations were performed. Animal sample size was chosen by the number of biological replicates necessary for ensuring statistical significance. The number of biological replicates is provided in the Method section. Since inbred mice with similar genetic background were selected as animal models, the chosen sample sizes are sufficient.
Data exclusions	No data exclusion.
Replication	Animal experiments were performed on biological replicates following identical procedures to verify the reproducibility of the experimental findings. All independent attempts at replication were successful.
Randomization	All samples/organisms were randomly allocated into experimental groups.
Blinding	The investigators were not blinded to group allocation during data collection and analysis but all assays were performed at the same time for all groups of a given experiment. Since all conditions were subjected to the same analyses, knowledge of group assignment will unlikely affect the behaviour in the trial and their responses to subjective outcome measures.

Reporting for specific materials, systems and methods

We require information from authors about some types of materials, experimental systems and methods used in many studies. Here, indicate whether each material, system or method listed is relevant to your study. If you are not sure if a list item applies to your research, read the appropriate section before selecting a response.

Materials & experimental systems

n/a	Involved in the study	
☒	☐	Antibodies
☐	☒	Eukaryotic cell lines
☒	☐	Palaeontology and archaeology
☐	☒	Animals and other organisms
☒	☐	Human research participants
☒	☐	Clinical data
☒	☐	Dual use research of concern

Methods

n/a	Involved in the study	
☒	☐	ChIP-seq
☐	☒	Flow cytometry
☒	☐	MRI-based neuroimaging

Eukaryotic cell lines

Policy information about cell lines

Cell line source(s)	MCF7 cells, EMT6 cells, HepG2 cells and LO2 cells were purchased from ATCC.
Authentication	All cell lines were authenticated by short tandem repeat (STR) profiling analysis.
Mycoplasma contamination	All cell lines tested negative for mycoplasma contamination.
Commonly misidentified lines (See ICLAC register)	No commonly misidentified cell lines were used in the study.

Animals and other organisms

Policy information about studies involving animals; ARRIVE guidelines recommended for reporting animal research

Laboratory animals	Female BALB/c mice (~4 weeks) were purchased from the Animal Experiment Center of Southern Medical University and raised in a sterile mouse house. The mice were maintained with free access to food and water in an environment of ambient temperature (~23 +/- 3 °C), 40~70% humidity and 12 h light/dark cycles.

Wild animals	No wild animals were used in this study.
Field-collected samples	No field-collected samples were used in this study.
Ethics oversight	All animal experiments comply with the Guidelines for the Care and Use of Laboratory Animals issued by the National Institutes of Health (NIH) of South China Normal University, and all animal experiments have been approved by the Animal Ethics Committee of South China Normal University.

Note that full information on the approval of the study protocol must also be provided in the manuscript.

Flow Cytometry

Plots

Confirm that:

☒ The axis labels state the marker and fluorochrome used (e.g. CD4-FITC).

☒ The axis scales are clearly visible. Include numbers along axes only for bottom left plot of group (a 'group' is an analysis of identical markers).

☒ All plots are contour plots with outliers or pseudocolor plots.

☒ A numerical value for number of cells or percentage (with statistics) is provided.

Methodology

Sample preparation	MCF7 cells were seeded in 6-well plates and cultured for 24 h until all cells were fully attached. The cells were treated with samples for 4 h and recultured in fresh medium. The plate was then irradiated under laser for 3 minutes. Then, the cells were washed with cold PBS, harvested and analyzed immediately using flow cytometry.
Instrument	CytoFLEX, Beckman Coulter, USA.
Software	FCS Express
Cell population abundance	The cell population abundance could not been determined because the use instrument was an analytical flow cytometry without sorting system. During sample measurements, the initial gate was used to ensure a cell count of 10,000 cells was collected for a relevant cell population.
Gating strategy	Compensation was used wherever necessary.

☒ Tick this box to confirm that a figure exemplifying the gating strategy is provided in the Supplementary Information.
